# Insights into diagnostic errors in endocrinology: a prospective, case-based, international study

**DOI:** 10.1186/s12909-023-04927-5

**Published:** 2023-12-08

**Authors:** Jessica Frey, Leah T. Braun, Laura Handgriff, Benjamin Kendziora, Martin R. Fischer, Martin Reincke, Laura Zwaan, Ralf Schmidmaier

**Affiliations:** 1 Medizinische Klinik und Poliklinik IV, University Hospital, Ludwig-Maximilians-University Munich, Ziemssenstr. 5, 80336 Munich, Germany; 2 Department of Dermatology and Allergology, University Hospital, LMU Munich, Munich, Germany; 3 Institute of Medical Education, University Hospital, LMU Munich, Munich, Germany; 4 Erasmus MC iMERR (Institute of Medical Education Research Rotterdam), Rotterdam, Netherlands

**Keywords:** Clinical reasoning, Diagnostic errors, Endocrinology, Internal medicine

## Abstract

**Background:**

Diagnostic errors in internal medicine are common. While cognitive errors have previously been identified to be the most common contributor to errors, very little is known about errors in specific fields of internal medicine such as endocrinology. This prospective, multicenter study focused on better understanding the causes of diagnostic errors made by general practitioners and internal specialists in the area of endocrinology.

**Methods:**

From August 2019 until January 2020, 24 physicians completed five endocrine cases on an online platform that simulated the diagnostic process. After each case, the participants had to state and explain why they chose their assumed diagnosis. The data gathering process as well as the participants’ explanations were quantitatively and qualitatively analyzed to determine the causes of the errors. The diagnostic processes in correctly and incorrectly solved cases were compared.

**Results:**

Seven different causes of diagnostic error were identified, the most frequent being misidentification (mistaking one diagnosis with a related one or with more frequent and similar diseases) in 23% of the cases. Other causes were faulty context generation (21%) and premature closure (17%). The diagnostic confidence did not differ between correctly and incorrectly solved cases (median 8 out of 10, *p* = 0.24). However, in incorrectly solved cases, physicians spent less time on the technical findings (such as lab results, imaging) (median 250 s versus 199 s, *p* < 0.049).

**Conclusions:**

The causes for errors in endocrine case scenarios are similar to the causes in other fields of internal medicine. Spending more time on technical findings might prevent misdiagnoses in everyday clinical practice.

**Supplementary Information:**

The online version contains supplementary material available at 10.1186/s12909-023-04927-5.

## Background

Diagnosing patients is a key competence of physicians. Establishing a correct diagnosis is the basis to select the best treatment for the patient. Nonetheless, diagnostic errors in medicine are frequent [1] and can have serious consequences for patients and their health [2]. An estimate from the National Academy of Medicine stated that most people will experience at least one diagnostic error in their lifetime, sometimes with severe consequences [3]. Therefore, more efforts into understanding the nature of diagnostic errors are crucial in order to reduce their occurrence and develop effective interventions. There is consensus amongst researchers that diagnostic errors are caused by both system and cognitive factors [2, 4]. Cognitive factors are considered the most common factor [2, 4–6]. According to Graber et al. they account for 74% of errors [4] and mainly occurred due to faulty synthesis, faulty data gathering, and faulty knowledge [4, 7]. Furthermore, errors often occur in the patient-physician encounter, including history taking and physical examination [6]. There is not yet consensus regarding the type of cognitive errors causing misdiagnosis. While some studies have suggested that cognitive biases (short cuts in the reasoning process) are the most common [4], others suggest that a lack of knowledge is the more important underlying factor [8–10]. Most of those studies involved a retrospective analysis of real clinical cases, which are sensitive to hindsight bias and may impact the physician´s critical assessment [11].

Clinical Reasoning – the ability to solve clinical cases – is not a general problem-solving skill [12] but it is case-specific. Therefore, it can be assumed that the kind of clinical encounters chosen for a study will influence the frequency and nature of diagnostic errors. One widely accepted theory explaining the cognitive processes in clinical reasoning is the dual processing theory [13]. Cognitive processes are controlled by two systems: System I, which is intuitive, fast and automatic, and System II, which is analytical and logical. Depending on the clinical experience and the familiarity with a specific clinical case, a physician will primarily use system I (for routine cases) or system II (for more unusual cases).

The endocrine field contains some common diseases, affecting millions of people each year, such as endocrine hypertension, diabetes mellitus or osteoporosis, but also very rare diseases (such as Cushing’s syndrome or pheochromocytoma), some of them potentially fatal, if misdiagnosed [14]. While content specific endocrine knowledge is often important for a correct diagnosis, patients with endocrine diseases often first present in a general practice or in general internal medicine. It is unclear how precisely physicians in general internal medicine or general practice can diagnose endocrine more umcommon cases and whether they are able to correctly identify “red flags”. We, therefore, chose to focus on endocrine cases in this study. Specifically, we developed a mix of cases that included diseases that are known to be commonly underdiagnosed although they are quite frequent, such as primary aldosteronism [15, 16] and hyponatremia, and potentially life-threatening, rare diagnoses that require quick diagnosis (Cushing’s syndrome, pheochromocytoma and Addison’s disease).

In order to study the reasoning process of general practitioners and general internists, we conducted a study in which participants prospectively solved endocrine cases in a virtual setting. The aim was to analyze the cognitive causes of diagnostic errors in for the participants unusual cases and to identify differences between correctly and incorrectly solved cases in the field of endocrinology.

## Materials and methods

### Design and participants

From August 2019 until January 2020, 24 physicians practicing internal medicine or general medicine completed a total of 111 simulated online clinical cases. The cases were all endocrine, however the participants were unaware of this, they were only informed that they were internal medicine cases.

The participants were chosen amidst one specific criterion, they had to be a physician practicing internal medicine, this included all subspecialties of internal medicine, as well as general medicine, excluding solely those practicing endocrinology, as it was expected that endocrinologists would make less errors, due to their better knowledge of endocrinological diseases and the goal of this study was to analyze as many diagnostic errors as possible. Apart from that all physicians practicing internal and or general medicine were included, regardless of their level of working experience, age or origin. The participants were recruited mainly through the listserv of the SIDM (society to improve diagnosing in medicine), as well as through flyers in the LMU (Ludwig MaximiIians University) hospital, as well as through directly contacting physicians from university clinics or general practitioner practices, using contact details provided on the respective websites. The participations did not receive a financial incentive.

### Case development

The cases were all written by one author (JF) based on real patient cases. Three resident and attending physicians specialized in endocrinology reviewed the cases. In an initial pilot study there were ten cases, completed by four physicians practicing internal medicine. The responses of the pilot were excluded from the data analysis. The aim of the pilot was to test the cases and the feasibility of the study. The five cases where most errors were made in the pilot, hence the most difficult, were selected for the actual study. Two cases with frequent diseases and three cases with very rare diseases were chosen for the study (Table 1). All cases are shown in the supplement.

**Table 1:** Overview of cases and contents of patient file

Overview of cases (correct diagnosis)	Prevalence of the disease	Frequency of the disease	Contents of patient file (identical for each case)
1. Primary aldosteronism	between 3.2–12.7% of patient with hypertension in primary care [17]	Frequent	Basic laboratory measures Specialized endocrine laboratory measures Ultrasound - abdominal Ultrasound - neck/thyroid ECG Chest X-Ray Spinal X-Ray MRI head 24 h blood pressure Urine analysis
2. Ectopic Cushing’s syndrome (paraneoplastic due to small cell lung cancer (SCLC))	between 0.7–2.4 to 0.2–5.0 per million people per year (prevalence of all subtypes of Cushing’s syndrome, about 10–20% of these are of ectopic origin) [18, 19]	Rare	
3. M. Addison (Addison’s disease)	about 80 per million people [20], other forms of adrenal insufficiency are more common	Rare	
4. SIADH (Syndrome of Inadequate Antidiuretic Hormone Secretion) (due to medication with citalopram)	hyponatremia is present in 15–30% of hospitalized patients, 1/3 of them suffer from SIADH [21]	Frequent	
5. Pheochromocytoma	in the US 500 to 1600 cases per year [22]	Rare	

### Study procedure

Participants first completed a sociodemographic questionnaire. Subsequently they diagnosed the five simulated internal medicine (endocrine) clinical cases (Table 1) on the online based platform CASUS [23]. This platform (details are shown in the supplement Fig. 1) enables the following of different steps of the diagnostic process. Each clinical case consisted of a patient history, a detailed physical examination and technical findings, i.e. results from laboratory and imaging, in the patient file (Table 1 for contents). The information in the history taking and physical examination consisted of age, gender, body mass index (BMI), vital parameters (blood pressure, heart rate, respiratory rate, body temperature) pre-existing illnesses, history of alcohol and nicotine consumption, cardiovascular, abdominal, lung and lymph node examination, a neurological examination, and their general and nutritional state. They were instructed to only look at the technical findings they deemed useful or essential to finding the correct diagnosis, in order to simulate the limited resources such as technical examinations and financial means in medical practice. However, the number of technical examinations that could be seen was not restricted. The number of technical findings viewed was recorded on the platform. Participants then had to state a diagnosis for each case, including an explanation and also had to indicate their diagnostic confidence (on a scale of 1–10, where 1 was not confident at all and 10 was very confident). They were able to switch between cases as they wished and were instructed to spend – very roughly - about 30 min on all cases to simulate the scarcity of time in medical practice, hence the time expenditure per case was measured. However, the time on task was not restricted.

### Content and statistical analysis

The content and the diagnostic steps were analyzed as described in detail in a previous study regarding diagnostic errors made by students [24]. The CASUS platform allows for gathering data prospectively and then analyze the physician’s diagnostic skills and diagnostic process. In addition, the technical findings participants looked at and how much time they spent on each finding was monitored. This, along with the explanation as to why physicians chose a diagnosis, helped understand in which part of the diagnostic process errors occurred.

The causes of diagnostic errors were ascribed to one error cause based on an already published classification, which was developed as an adaption of Graber’s diagnostic errors classification [24]. The seven error categories are: inadequate knowledge base, inadequate diagnostic skills, faulty context generation, overestimating/underestimating, faulty triggering, misidentification and premature closure. For a detailed description on the development of these error categories see Braun et al. [24]. More details on how the errors were assigned to a category can be found in the supplement (supplement Table 1).

Diagnostic explanations were qualitatively analyzed [25]. Each diagnostic error was assigned to one category (Table 2). We assigned each case only to one category by choosing the predominant error that finally caused the misdiagnosis. One investigator (JF) coded all errors. A second rater (LB) also independently coded all errors and explanations. The interrater coefficient analyzed with Cohens Kappa was 0.79. The causes of misdiagnoses were quantitatively assessed.

**Table 2:** Frequency of causes for diagnostic errors

Cause	Definition	Example (given diagnostic explanation)	Frequency N = 52 (100%)
Misidentification	One diagnosis is mistaken for another	„Pathological dexamethasone-suppression-test. Already diabetes and hypertension as a result of the disease” (Diagnosed pituitary Cushing’s syndrome. Correct diagnosis: Ectopic Cushing’s syndrome)	12 (23%)
Faulty context generation	Lack of awareness of relevant condition	“either it’s a harmless gastrointestinal infection (but she doesn’t have diarrhea and the duration doesn’t match), because of the age and the course of several days it could also be a pregnancy” (diagnosed pregnancy in a patient with SIADH)	11 (21%)
Premature closure	Does not consider other diagnoses	“The patient’s age and the absence of organic data in the central nervous system” (Physician diagnosed migraine instead of primary hyperaldosteronism without looking at any of the laboratory results or the imaging)	9 (17%)
Lack of knowledge	Insufficient knowledge	„arterial hypertension without evidence of an endocrine cause” (diagnosed arterial hypertension instead of primary hyperaldosteronism, looked at the laboratory results)	8 (15%)
Faulty triggering	Wrong conclusions	“Psychotropic drugs often cause SIADH“ (physician diagnosed gastroenteritis instead of SIADH, although he gave this correct explanation)	6 (12%)
Overestimating/ Underestimating	Focus to closely, ignores important aspects	„HbA1c 7%, adynamia“ (diagnosed diabetes in a patient with an adrenal crisis due to Addison’s disease)	5 (10%)
Lack of diagnostic skills	Insufficient skills e.g. to interpretate an x-Ray	“ECG” (diagnosed hear block in a patient with pheochromocytoma who did not have a heart block in his ECG)	1 (2%)

Diagnoses were binary coded as correct or incorrect. Cases with correct and incorrect diagnosis were compared regarding the time spent on a case, number of technical findings viewed, and diagnostic confidence.

Means and standard deviations were calculated to describe continuous variables. Absolute counts and percentage shares were applied for describing categorical variables. *P*-values of equal or less than 5% were considered significant. Statistical analysis was performed in SPSS 27. Differences between groups were tested by the Mann-Whitney-U-Test due to a lack of normal distribution.

## Results

### Participants

24 (18 male, 6 female) participants completed 111 cases in total, 9 cases were not completed. Their mean age was 45 years (SD ± 15.6). Most participants were general practitioners (36%) or working in general internal medicine (21%) whereas the remaining participants were specialized in other fields of internal medicine (Table 3). Their working settings included both hospitals and practices.

**Table 3:** Sociodemographic overview of participants (n = 24)

**Age (years)**	• 45.4 ± 15.6
**Gender**	• Male: 18 (75%) • Female: 6 (25%)
**Country of origin**	• Germany:15 (63%) • USA: 5 (21%) • Others: 4 (16%)
**Work Setting**	• University hospital: 14 (58%) • Practice: 5 (21%) • Private clinic/district clinic: 3 (13%) • Other (not specified): 2 (8%)
**Work experience (years)**	• 1–6 years (cohort 1: less experienced): n = 10 (42%) • > 6 years (cohort 2: more experienced)’n = 14 (58%)
**Specialization**	• General medicine: 9 (36%) • Internal medicine: 5 (21%) • Nephrology 3 (13%) • Cardiology: 3 (13%) • Other specializations: 4 (16%)
**Further obligations**	• Medical education & research: 14 (58%) • Only medical education: 6 (25%) • None: 4 (17%)

### Results of the error analysis: frequency, nature and distribution of errors

The physicians misdiagnosed 52 out of 111 times, with a total error frequency of 47%. The mean time expenditure per case was 9 min and 48% of the technical findings were viewed. The frequencies of different causes for errors is shown in Table 2. Overall, the most common error type in all completed cases were misidentification and faulty context generation. Amongst the five cases, case 3 (Addison’s disease) had the lowest error frequency (33%) and case 2 (ectopic Cushing’s) had the highest (73%). The leading cause of diagnostic error differed from case to case (Table 4). In cases with rare diseases, a lack of knowledge was not more frequently a cause of errors compared to the cases with more frequent diseases.

**Table 4:** Kind of incorrect diagnoses

	Case 1: Primary aldosteronism	Case 2: Ectopic Cushing’s syndrome	Case 3: Addison’s disease	Case 4: SIADH	Case 5: Pheochromocytoma
Error rate (%)	54%	73%	33%	36%	36%
Mean percentage of findings viewed (%)	52%	52%	44%	40%	54%
Number of wrong diagnoses	13	16	7	8	8
Main cause for cognitive errors	Inadequate knowledge (N = 6)	Misidentification (N = 8)	Overestimation (N = 3)	Faulty context generation (N = 4)	Premature closure (N = 6)
**Frequency of certain misdiagnoses**					
	(primary) arterial hypertension (N = 8)	cushing’s syndrome (N = 2)	diabetes mellitus (N = 3)	pregnancy (N = 2)	atrial fibrillation (N = 5)
	pheochromocytoma (N = 3)	hypothyroidism (N = 3)	syncope (N = 1)	toxicity (N = 1)	heart Block (N = 1)
	secondary arterial hypertension (N = 1)	cushing’s disease (N = 6)	av-block, first grade (N = 1)	anorexia (N = 1)	lymphoma (N = 1)
	migraine (N = 1)	depression (N = 2)	pituitary tumor (N = 1)	gastroenteritis (N = 1)	coronary heart disease (N = 1)
		lung cancer (N = 1)	polyglandular autoimmune syndrome (N = 1)	pituitary insufficiency (N = 2)	
		pituitary tumor (N = 1)		hyponatremia (N = 1)	
		adrenal cushing’s syndrome (N = 1)			

### Misdiagnoses in different diseases

The kind of misdiagnoses were evaluated to explore possible patterns (Table 4). In all five cases, misdiagnoses were mostly very common diagnoses. For example, *primary hyperaldosteronism* was most often diagnosed as primary arterial hypertension. The misidentification with other rare diseases (pheochromocytoma) was less common. In the case of *ectopic Cushing’s syndrome*, physicians made the most misdiagnoses, mostly due to the fact, that they did not diagnose accurately enough: A lot of physicians diagnosed a Cushing’s syndrome, but did not classify it as an ectopic Cushing’s syndrome (but rather as Cushing’s syndrome, Cushing’s disease or pituitary tumor). When determining the error types for this case, various participants overlooked the lung mass in the thoracic X-ray, which was essential to finding the correct diagnosis. In the case *SIADH*, other common diagnoses were stated instead of the correct one (pregnancy, gastroenteritis). In the pheochromocytoma case, one of the clinical signs – tachycardia and atrial fibrillation – was stated as final diagnosis, although it was a symptom of the underlying disease.

### Correctly and incorrectly solved cases

The time per case, the time spent on patient’s history or physical examination did not differ between correctly and incorrectly solved cases (Table 5). Furthermore, the number of technical findings that were looked at by the physicians did not differ between the cases, but in correctly solved cases, physicians spent more time on these technical findings.

**Table 5:** Comparison of correctly and incorrectly solved cases

Cohort	Correctly solved cases	Incorrectly solved cases	
**N**	N = 60	N = 51	
**Median diagnostic confidence** *(1–10 points with 1 being the lowest confidence and 10 highest)*	8 (7–9)	8 (6–9)	
**Median time expenditure per case** *(min)*	5.9 (3.8–7.4)	4.8 (2.6–7.9)	
**Median time expenditure per patient’s history** *(sec)*	28 (19–53)	33 (23–50)	
**Median time expenditure per physical examination** *(sec)*	46 (32–72)	54 (33–79)	
**Median time expenditure per technical examinations** *(sec)*	250 (179–332)	199 (86–284)	
**Median percentage of findings viewed** *(N of a maximum of 10)*	5 (3–7)	4 (2–6)	

Shown as median and ranges. Test used: Mann-Whitney-Test

The diagnostic confidence was very high both in the correctly and incorrectly solved cases (median diagnostic confidence: 8 out of 10).

## Discussion and conclusion

### Causes of errors in endocrinology

We were able to distinguish seven different cognitive error types. Overall, the most common error categories were misidentification, premature closure and faulty context generation. These findings are in line with previous studies [2, 4]:

In this study, all misdiagnoses were assigned only to one category, so that a single root cause of the nature of the error was determined. It is a strength of the study that the reflections of the physicians are available. This is an insight that most studies, particularly retrospective ones, do not have. By analyzing the explanations, we could determine the cause of an error with more certainty.

However, in other studies, it was also described that errors are often multifactorial [4, 26]. Hence it could be that certain error causes are interdependent. For instance, if physicians have a cognitive bias due to overconfidence, such as stereotyping based on certain information of the patient, this could lead to premature closure, where they do not look closely at further technical findings, as they have already come to a premature diagnosis, hence leading to over- and underestimating of certain information. Other studies suggest lack of diagnostic skills (e.g. interpretation of imaging) may even be an underlying factor of premature closure [14].

Not all diagnostic errors in endocrinology are of the same severity. For example, in the case of a patient with ectopic Cushing’s syndrome, the most errors occurred. However, the most common misdiagnosis – Cushing’s syndrome - is not completely incorrect but just imprecise. In everyday clinical practice, it is important for general practitioners and physicians working in general internal medicine to identify the correct specialty from which the disease could originate, so that the patient can be transferred to a specialist. An endocrinologist will be able (in the majority of cases) to classify this patient correctly as a patient having ectopic Cushing’s syndrome. In clinical practice, this incorrectness will possibly not harm the patient. However, in other cases with rare diseases such as pheochromocytoma the misdiagnoses might have much severer consequences. This should be kept in mind when we analyze diagnostic errors: Not every error will harm a patient. Therefore, just the frequency of errors is not critical but the causes of the errors, the kind of misdiagnoses and the therapy decisions based on those errors. A wrong diagnosis can nevertheless result in a correct treatment as already shown [27].

### Correctly and incorrectly solved cases

Interestingly, we observed very little differences regarding time on task or number of technical findings viewed between correctly and incorrectly solved cases, which suggests that there are no major differences in the reasoning process of correct and incorrect diagnosis, but it likely depends more on the knowledge of physicians. It is notable that in correctly solved cases, physicians spent more time on technical examinations such as laboratory results and imaging. Spending more time on these findings might prevent premature closure and faulty context generation. This finding is different from several previous studies where correct diagnoses were often based on a faster diagnostic process [28, 29]. One explanation could be that in this study the cases were endocrine and therefore outside the main clinical expertise of participants. They may have led to a closer review of the technical findings in cases where physicians were correct. An interesting finding is that the levels of confidence in the diagnosis were rather high in both correct and incorrect diagnosis. The fact that physicians have poor calibration between confidence and accuracy is in line with previous studies [30, 31]. However, the overall confidence levels seem very high, reflecting a strong overconfidence of physicians in this study. Also, a low learning motivation can be associated with overconfidence as recently shown [32], which should be kept in minding in previous studies.

### Strengths and limitations

Strengths of this study are the prospective design and the comprehensive analysis of the causes of errors enabled by the study platform CASUS. Moreover, as many of the participants were general practitioners, who patients often initially consult, this study simulates the primary care situation. Also, we had a multicentric, international approach, which is another advantage of the study.

The limitations of the study include the limited sample size, as a sample of 24 physicians may not accurately represent the broader population of healthcare professionals. It has to be considered that our sample size may not accurately represent the broader population of healthcare professionals. The selection of the participants might be influenced by availability or interest. However, as quite a large number of cases was analyzed and we were able to draw valid conclusions of that which might be addressed in upcoming larger studies.

However, we qualitatively analyzed 111 cases, which is quite extensive. Furthermore, in this study, the CASUS platform, although realistic, does present an artificial setting. Therefore, we cannot be sure that the findings apply to clinical practice. It is a constraint of the study, whether the findings can truly be seen to represent cognitive processes in genuine clinical encounters. For example, participants could not profit from discussions with colleagues, which could help find the correct diagnosis [33]. Additionally, in everyday clinical practice, errors can be multifactorial and more unpredictable as more variables influence the outcome. However, even in everyday practice there are errors only caused by cognitive factors (see Graber et al.) and at least for those instances, the results of the study seem applicable. Additionally, we focused on difficult endocrinological cases, therefore, our results regarding the causes of errors might not be applicable for more common and less difficult endocrinological encounters. However, the cases chosen for a study will always influence the results regarding diagnostic errors as clinical reasoning is indeed case-specific. Therefore, this is a general limitation not only affecting our study. A further limitation is that we may not capture the full spectrum of endocrinological cases encountered in clinical practice. Also, there is the possibility of certain participants being familiar with the casus interface, and hence allowing them to more easily navigate through the findings, however the instructions on how to navigate through the findings are easily comprehendible.

### Outlook and conclusion

To the best of our knowledge, errors in endocrinology made by physicians were analyzed for the first time in a controlled setting in this prospective study. Predominant causes for errors in this specialty include misidentification, faulty context generation, premature closure and a lack of knowledge which is in line with previous findings on causes of diagnostic error. The process of correctly diagnosed cases did not differ much from the incorrectly diagnosed cases. This suggests that it is not the reasoning process that is different in cases with and without errors, but that it is the content specific knowledge that plays the most important role. Therefore, practice with a large variety of clinical cases (including endocrine cases) in continuing medical education seems advantageous for error reduction.

### Electronic supplementary material

Below is the link to the electronic supplementary material.

**Supplementary Material 1: Supplement Figure 1:** The CASUS surface. **Supplement Table 1:** The different error categories with explanation. **Supplement: Cases** Case 1: Morbus Conn. Case 2: Ectopic Cushing’s syndrome(paraneoplastic due to small cell lung cancer). Case 3: Morbus Addison. Case 4: SIADH (caused by medication, due to citalopram). Case 5: Pheochromocytoma

## Data Availability

The datasets used and/or analysed during the current study are available from the corresponding author on reasonable request.
