# Supplementary material for: Insights into diagnostic errors in endocrinology: a prospective, case-based, international study
Source: BMC Med Educ. 2023 Dec 8;23:934. doi: 10.1186/s12909-023-04927-5 (PMC10709946; doi:10.1186/s12909-023-04927-5)
Supplement: Supplementary file 1 — Supplementary Material 1: Supplement Figure 1: The CASUS surface. Supplement Table 1: The different error categories with explanation. Supplement: Cases Case 1: Morbus Conn. Case 2: Ectopic Cushing’s syndrome(paraneoplastic due to small cell lung cancer). Case 3: Morbus Addison. Case 4: SIADH (caused by medication, due to citalopram). Case 5: Pheochromocytoma [file 12909_2023_4927_MOESM1_ESM.docx]

**Supplement**

**Supplement Figure 1: The CASUS surface**


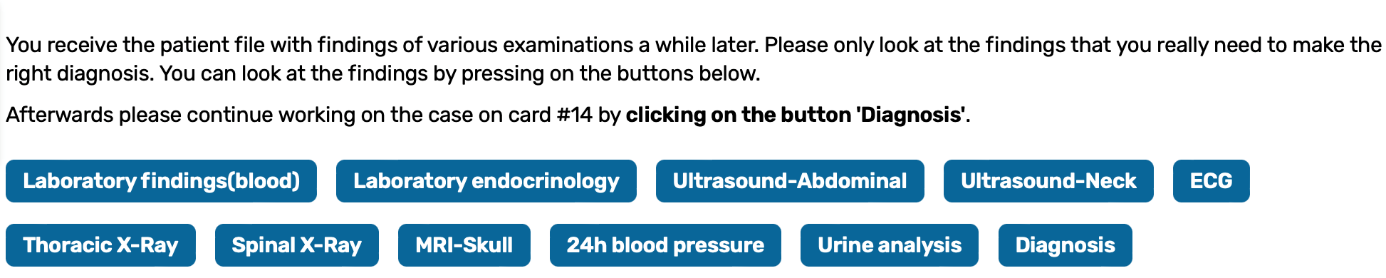


**Supplement Table 1: The different error categories with explanation**

| ***Category*** | ***Explanation*** | ***Distinction to similar categories*** |
| --- | --- | --- |
| *Misidentification* | *The physician mistakes one diagnosis for another, which is similar regarding clinical signs or pathophysiology.* | *-* |
| *Faulty context generation* | *The participant looked at the important clinical information but was not aware of important lab results or other diagnostic information.* | *In contrast to the category premature closure, the physician completed the data gathering process. In contrast to the category faulty triggering, the physician gave a wrong explanation for his final diagnosis.* |
| *Premature closure* | *The physicians did not look at all the relevant diagnostic information and draw direct conclusions from the history of the patient.* | *An error was only assigned to this category, it the data gathering was incomplete.* |
| *Lack of knowledge* | *The physician did look at all the relevant diagnostic information, e.g. lab results, but did not know when results were pathological.* | *The physician explicitly mentioned results as pathological or normal, although it was the opposite. Technical results (x-Ray or ECG) were excluded from this category.* |
| *Faulty triggering* | *The physician looked at all the important information, the diagnostic explanation was correct but still a wrong diagnosis was given.* | *In contrast to the category premature closure, the physician completed the data gathering process. In contrast to the category faulty context generation, the physician gave a correct diagnostic explanation.* |
| *Overestimating/ underestimating* | *The physician valued an incidental finding so high that a wrong diagnosis based on the incidental finding was given.* | *-* |
| *Lack of diagnostic skills* | *The physician obviously did not draw the correct conclusions from an x-Ray or ECG (if he or she described something that was not there or did not see a relevant/pathological feature).* | *The error was only assigned to that category, if the physician clearly misinterpreted a technical examination.* |

**Supplement: Cases**

**Case 1: Morbus Conn**

**Anamnesis:** The 25 year old Ms. S. introduces herself with a strong headache and dizziness, that occur every morning. These have been recurring throughout the last few weeks. Furthermore she mentions a tingling sensation, especially prominent on the skin of her fingers, occasional ringing in the ears and an extreme feeling of exhaustion. No pre-existing medical conditions are known of. Ms. S. does not take any medicine.  She states that she has been smoking a pack of cigarettes daily for the past 6 years (6 pack years) and that she consumes alcoholic beverages occassionally.

**Physical examination:**

In the physical examination you are able to collect the following findings:

Awake, approachable, 4-times oriented patient in average general condition and normal nutritional state(1,70 m, 58kg, BMI 20,1)

**Vital signs**: blood pressure 170/100 mmHg, heart rate 99/min, respiratory rate 15/min, body temperature(ear) 37,0°C

**Cardiovascular system**: heart sounds rhythmical and normal, no pathological heart sounds, no flow of noise above the carotides. No jugular veinous distensions, no peripheral edema, peripheral pulses (A. radialis both sides, A. dorsalis pedis both sides, A. tibialis posterior both sides) strongly palpable.

**Respiratory System:** No chest wall deformities. no lip cyanosis. no dullness on percussion, lung borders on both sides are displacable by 2 finger widths upon breathing. vesicular breath sounds, no crackles, weezing or stridor. Painless percussion of thoracic spine

**Abdomen**: inconspicious inspection lively abdominal sounds above all 4 quadrants, no abdominal flowing noises. soft abdominal wall, painless palpation, no resistance. normal percussion. Painless palpation of soft liver border upon inhalation, 11cm in the midclavicular line upon percussion. Spleen not palpable. No hernias. Painless kidney percussion. Painless percussion of lumbar spine.

**Throat**: Thyroid of soft consistency, movement upon swallowing, no knots palable

**Lymph nodes** : no enlarged cervical , axillary or inguinal lymph nodes palpable

**Neurological examination**

awake, fully conscious, no cognitiv abnormalities, adequate verbal response, no focal neurologic deficit

**Pupils**:  medium width on both sides, isocoric, prompt pupillary light response on both sides

**Motor activity**: no latent or manifested paresis and normal muscle tone

**Reflexes**: performable on both sides (patellar tendon reflix, achilles tendon reflex, biceps tendon reflex) without enlarged reflex zones, Babinski negative

**Sensibility**: all qualities intact on both sides

**Cranial nerves**: cranial nerves intact, inconspicious ocular motoric activity, no visual defect, no nystagmus

**Gait and coordination**: inconspicious according to age

**Case 2: Ectopic Cushing’s syndrome(paraneoplastic due to small cell lung cancer)**

**Anamnesis:** The 55 year old Mrs. Y. introduces herself with muscular weakness and fatigue. Moreover she mentions that she gained 8 kg in the last 2 months and that she has felt rather downcast recently. The following pre-existing medical conditions are known of: COPD. Spiriva is taken when needed. Mrs. Y. has been smoking a pack of cigarettes a day for the past 30 years(30 pack years); no consumption of alcohol.

**Physical examination:**

In the physical examination you are able to collect the following findings:

approachable patient in decreased general condition and obese nutritional state(1,60 m, 78kg, BMI 30,4)

**Vital signs**: blood pressure 160/100 mmHg, heart rate 90/min, respiratory rate 15/min, body temperature(ear) 36,9°C

**Cardiovascular system**: heart sounds rhythmical and normal, no pathological heart sounds, no flow of noise above the carotides. No jugular veinous distensions, no peripheral edema , peripheral pulses (A. radialis both sides, A. dorsalis pedis  both sides, A. tibialis posterior both sides) palpable.

**Respiratory System**: No chest wall deformities. no lip cyanosis. hypersonor sound upon percussion,low standing of diaphragm.  dry wheezing sound when breathing.painful percussion of thoracic spine

**Abdomen**: inconspicious inspection. lively abdominal sounds above all 4 quadrants, no abdominal flowing noises. soft abdominal wall, painless palpation, no resistance. normal percussion. Painless palpation of soft liver border upon inhalation, 11cm in the midclavicular line upon percussion. Spleen not palpable. No hernias. Painless kidney percussion. Painless percussion of lumbar spine.

**Throat**: Thyroid of soft consistency, movement upon swallowing, no knots palable

**Lymph nodes**: no enlarged cervical , axillary or inguinal lymph nodes palpable

**Neurological examination**

awake, fully conscious, gloomy, slight cognitiv limitation, adequate verbal response, no focal neurologic deficit

**Pupils**:  medium width on both sides, isocoric, prompt pupillary light response on both sides

**Motor activity**: no latent or manifested paresis and normal muscle tone

**Reflexes**: performable on both sides (patellar tendon reflix, achilles tendon reflex, biceps tendon reflex) without enlarged reflex zones, Babinski negative

**Sensibility**: all qualities intact on both sides

**Cranial nerves:** cranial nerves intact, inconspicious ocular motoric activity, no visual defect, no nystagmus

**Gait and coordination:** inconspicious according to age

**Case 3: Morbus Addison**

**Anamnesis:** The 33 year old Mr. F. has been experiencing a feeling of weakness the past few days. Yesterday at work he even fainted for a short while. This has never occured before. No pre-existing medical conditions are known of and Mr. F. does not take any medication. The patient does not smoke and seldomly consumes alcohol.

**Physicial examination:**

In the physical examination you are able to collect the following findings: awake, approachable, fully oriented patient in good general condition and normal nutritional state (1,85 m, 80 kg, BMI 23)

**Vital signs**: blood pressure 112/68 mmHg, heart rate 80/min, respiratory rate 14/min, body temperature(ear) 36,6°C

**Cardiovascular system**: heart sounds rhythmical and normal, no pathological heart sounds, no flow of noise above the carotides. No jugular veinous distensions, no peripheral edema , peripheral pulses (A. radialis both sides, A. dorsalis pedis  both sides, A. tibialis posterior both sides) strongly palpable.

**Respiratory System:** No chest wall deformities. no lip cyanosis. no dullness on percussion, lung borders on both sides are displacable by 2 finger widths upon breathing. vesicular breath sounds, no crackles, weezing or stridor. painless percussion of thoracic spine-

**Abdomen**: inconspicious inspection. lively abdominal sounds above all 4 quadrants, no abdominal flowing noises. soft abdominal wall, painless palpation, no resistance. normal percussion. Painless palpation of soft liver border upon inhalation, 11cm in the midclavicular line upon percussion. Spleen not palpable. No hernias. Painless kidney percussion. Painless percussion of lumbar spine.

**Throat**: Thyroid of soft consistency, movement upon swallowing, no knots palable

**Lymph nodes**: no enlarged cervical , axillary or inguinal lymph nodes palpable

**Neurological examination**

awake, but exhausted, fully oriented and conscious, adequate verbal response, no focal neurologic deficit

**Pupils**: medium width on both sides, isocoric, prompt pupillary light response on both sides

**Motor activity**: no latent or manifested paresis and reduced muscle tone

**Reflexes**: performable on both sides (patellar tendon reflix, achilles tendon reflex, biceps tendon reflex) without enlarged reflex zones, Babinski negative

**Sensibility:** all qualities intact on both sides

**Cranial nerves**: cranial nerves intact, inconspicious ocular motoric activity, no nystagmus

**Gait and coordination**: inconspicious according to age

**Case 4:** SIADH( caused by medication, due to citalopram)

**Anamnesis:** The 31 year old Mrs. S.. introduces herself with nausea and vomiting, which have been present since the last few days. The only known pre-existing medical condition is depression, which is treated with Citalopram. Apart from this she does not take any medication. Mrs. S. does not drink alcohol or smoke.

**Physical examination:**

In the physical examination you are able to collect the following findings: awake, approachable, lethargic, 4-times oriented patient in decreased general condition and decreased nutritional state (1,70 m, 50 kg, BMI 17)

**Vital signs**: blood pressure 90/60 mmHg, heart rate 80/min, respiratory rate 14/min, body temperature(ear) 37,0°C

**Cardiovascular system**: heart sounds rhythmical and normal, no pathological heart sounds, no flow of noise above the carotides. No jugular veinous distensions, no peripheral edema , peripheral pulses (A. radialis both sides, A. dorsalis pedis  both sides, A. tibialis posterior both sides) strongly palpable.

**Respiratory System**: No chest wall deformities. no lip cyanosis. no dullness on percussion, lung borders on both sides are displacable by 2 finger widths upon breathing. vesicular breath sounds, no crackles, weezing or stridor. Painless percussion of thoracic spine.

**Abdomen**: inconspicious inspection lively abdominal sounds above all 4 quadrants, no abdominal flowing noises. soft abdominal wall, painless palpation, no resistance. normal percussion. Painless palpation of soft liver border upon inhalation, 11 cm in the midclavicular line upon percussion. Spleen not palpable. No hernias. Painless kidney percussion. Painless percussion of lumbar spine.

**Throat**: Thyroid of soft consistency, movement upon swallowing, no knots palable

**Lymph nodes**: no enlarged cervical , axillary or inguinal lymph nodes palpable

**Neurological examination**

awake, fully conscious, adequate verbal response, no focal neurologic deficit

**Pupils**:  medium width on both sides, isocoric, prompt pupillary light response on both sides

**Motor activity:** no latent oder manifested paresis and  normal muscle tone

**Reflexes:**performable on both sides (patellar tendon reflix, achilles tendon reflex, biceps tendon reflex) without enlarged reflex zones, Babinski negative

**Sensibility:**all qualities intact on both sides

**Cranial nerves:** cranial nerves intact, inconspicious ocular motoric activity, no nystagmus

**Gait and coordination:**inconspicious according to age

**Case 5: Pheochromocytoma**

**Anamnesis:** The 51 year old Mr. F. introduces himself with recurrent headaches. He has also noticed heart palpitations and increased sweating for the past week. He also mentions having pain in the left chest sometimes and having lost 4 kg. No pre-existing medical conditions are known of. Mr. F. does not smoke and only consumes alcohol on special occasions.

**Physical examination:**

In the physical examination you are able to collect the following findings: awake, approachable,  4-times oriented patient in decreased general condition and average nutritional state (1,85 m, 80 kg, BMI 23)

**Vital signs:** blood pressure 140/95 mmHg, heart rate 93/min, respiratory rate 15/min, body temperature(ear) 37,5°C

**Cardiovascular system**: heart sounds rhythmical and normal, no pathological heart sounds, no flow of noise above the carotides. No jugular veinous distensions, no peripheral edema , peripheral pulses (A. radialis both sides, A. dorsalis pedis  both sides, A. tibialis posterior both sides) easily palpable.

**Respiratory System:** No chest wall deformities. no lip cyanosis. no dullness on percussion, lung borders on both sides are displacable by 2 finger widths upon breathing.  vesicular breath sounds, no crackles, weezing or stridor. painless percussion of thoracic spine

**Abdomen:** inconspicious inspection lively abdominal sounds above all 4 quadrants, no abdominal flowing noises. soft abdominal wall, painless palpation, no resistance. normal percussion. Painless palpation of soft liver border upon inhalation, 11cm in the midclavicular line upon percussion. Spleen not palpable. No hernias. Painful kidney percussion. Painless percussion of lumbar spine.

**Throat:** Thyroid of soft consistency, movement upon swallowing, no knots palable

**Lymph nodes:** no enlarged cervical, axillary or inguinal lymph nodes palpable

**Neurological examination**

awake,  fully conscious, adequate verbal response, no focal neurologic deficit

**Pupils:** medium width on both sides, isocoric, prompt pupillary light response on both sides

**Motor activity:**no latent oder manifested paresis and normal muscle tone

**Reflexes:** performable on both sides (patellar tendon reflex, achilles tendon reflex, biceps tendon reflex) without enlarged reflex zones, Babinski negative

**Sensibility:** all qualities intact on both sides

**Cranial nerves:** cranial nerves intact, inconspicious ocular motoric activity, no nystagmus

**Gait and coordination:**inconspicious according to age
